# On the Effects of Scale for Ecosystem Services Mapping

**DOI:** 10.1371/journal.pone.0112601

**Published:** 2014-12-30

**Authors:** Adrienne Grêt-Regamey, Bettina Weibel, Kenneth J. Bagstad, Marika Ferrari, Davide Geneletti, Hermann Klug, Uta Schirpke, Ulrike Tappeiner

**Affiliations:** 1 Planning of Landscape and Urban Systems, Swiss Federal Institute of Technology (ETH), Stefano-Franscini-Platz 5, 8093 Zurich, Switzerland; 2 U.S. Geological Survey, Geosciences and Environmental Change Science Center, P.O. Box 25046, MS 980, Denver, Colorado, 80225, United States of America; 3 Department of Civil, Environmental and Mechanical Engineering, University of Trento, Via Mesiano 77, 38123 Trento, Italy; 4 Interfaculty Department of Geoinformatics – Z_GIS, University of Salzburg, Schillerstr. 30, 5020 Salzburg, Austria; 5 Institute for Alpine Environment, EURAC research, Viale Druso 1, 39100 Bolzano, Italy; 6 Institute of Ecology, University of Innsbruck, Sternwartestr. 15, 6020 Innsbruck, Austria; University of Auckland, New Zealand

## Abstract

Ecosystems provide life-sustaining services upon which human civilization depends, but their degradation largely continues unabated. Spatially explicit information on ecosystem services (ES) provision is required to better guide decision making, particularly for mountain systems, which are characterized by vertical gradients and isolation with high topographic complexity, making them particularly sensitive to global change. But while spatially explicit ES quantification and valuation allows the identification of areas of abundant or limited supply of and demand for ES, the accuracy and usefulness of the information varies considerably depending on the scale and methods used. Using four case studies from mountainous regions in Europe and the U.S., we quantify information gains and losses when mapping five ES - carbon sequestration, flood regulation, agricultural production, timber harvest, and scenic beauty - at coarse and fine resolution (250 m vs. 25 m in Europe and 300 m vs. 30 m in the U.S.). We analyze the effects of scale on ES estimates and their spatial pattern and show how these effects are related to different ES, terrain structure and model properties. ES estimates differ substantially between the fine and coarse resolution analyses in all case studies and across all services. This scale effect is not equally strong for all ES. We show that spatially explicit information about non-clustered, isolated ES tends to be lost at coarse resolution and against expectation, mainly in less rugged terrain, which calls for finer resolution assessments in such contexts. The effect of terrain ruggedness is also related to model properties such as dependency on land use-land cover data. We close with recommendations for mapping ES to make the resulting maps more comparable, and suggest a four-step approach to address the issue of scale when mapping ES that can deliver information to support ES-based decision making with greater accuracy and reliability.

## Introduction

The increasing recognition that unsustainable growth in population, per-capita consumption, and related environmental impacts threatens both ecosystems and the services they provide to people calls for an operationalization of the ecosystem services (ES) concept [1]–[5]. Global, EU, and national policies such as the Convention on Biological Diversity, the EU Biodiversity Strategy, and 2012 U.S. Department of Agriculture-Forest Service Planning Rule (36 CFR 219) require ES quantification, valuation, and mapping to assist decision makers in managing areas supporting high biodiversity and ES provision [6]. Particularly when trade-offs must be made in allocating land or other resources to competing human uses, maps of ES under different scenarios can help identify synergies and trade-offs among the different ES, better supporting decision making that balances long-term protection of ecosystems and their services against short-term economic development goals, e.g. [7]–[10]. Studies mapping the supply of and demand for multiple ES from the global to the sub-national scale are increasingly common [11]–[16], and show that both the diverse approaches to model and map ES and the different scales of ES assessments can yield a wide range of metrics with results that differ at best and are incomparable at worst. If ES mapping is to effectively assist in spatial targeting of conservation and development activities, or in evaluating the benefits and costs of alternative policies [17], the effect of scale on the resulting ES maps should be addressed [18]–[20]. A full consideration of scale includes: (1) the cartographic scale, describing the ratio of real to mapped distance, (2) the spatio-temporal extent of the study area, (3) the grain, or finest spatial resolution within a given dataset, i.e. cell size, (4) the level of organization within the biotic hierarchy, (5) the resolution or the precision of measurement, the same as grain size in spatial context [21], and (6) the level of jurisdiction or aggregation of human institutions involved in the combined social-ecological system [22]. For this paper, our analysis focuses on the effects of cell size on the results of monetary and non-monetary ES estimates in mountain regions.

There is a long history of research on the effects of different aspects of scale, for example in geography [23]–[25] and ecology [26]–[28]. Model resolution and spatio-temporal extent are ideally chosen to correspond with their underlying biophysical and socioeconomic processes [29]. Some ecological processes are associated with a particular scale, while other processes may occur across multiple scales. Wiens [26] provide ecological examples of scale effects on patterns. For example, domestic cattle grazing in shortgrass prairie preferentially graze specific plants, but landscape-scale grazing of vegetation types is proportional to their coverage at that broader scale. Choosing the right scales thus means understanding these processes at varying spatial and temporal scales. Furthermore, when investigating socio-ecological systems, one cannot assume that ecological and social processes operate at the same scales, and linkages must often be developed to connect across scales [20], [30]–[33]. For instance, conventional resource management has often increased the potential for larger-scale disturbances and less predictable and less manageable environmental feedbacks. These feedbacks can have unpredictable, negative effects on ecosystems and societies that depend on the resources and services that ecosystems generate [34]. For example, instead of seeking to reduce local air pollution, taller smokestacks have been built as a remedy to air pollution. If that took place on a limited scale, the atmosphere’s capacity as a pollutant sink might not be exceeded. But when numerous local stacks were built, they caused an accumulation of sulfur compounds in the air that resulted in widespread regional acidification of aquatic ecosystems. Thus a small, localized disturbance was turned into a larger regional disturbance [34]. But while there is increasing recognition that different processes operate at different scales and that cross-scale dynamics should be considered in the studies of socio-ecological systems [29], [35]–[41], the issue of scale in ES quantification, valuation, and mapping has only recently been addressed. The MA (Millennium Ecosystem Assessment) [42] raised this issue by showing that some losses of ES were related to the mismatch between the scales of ecosystem processes and those at which governance institutions were effective, ranging from community level to international relationships [1]. Hein et al. [32] showed that ES estimates can change considerably depending on the stakeholders and the scales of their associated institutions. However, a quantitative understanding of the diverse scaling issues associated with ES mapping has not yet been achieved. It is unclear, for instance, whether fine or coarse resolution input data should be expected to yield greater or lesser modeled ES estimates. Konarska et al. [43] found 198% greater monetary ES values using value transfer when paired with fine resolution (30 m) relative to coarse resolution input data (1 km). They attributed this to the loss of high-value land cover types that cover a small spatial extent (e.g., wetlands) when land cover is classified at a coarse spatial resolution. This aligns with the findings of Turner et al. [27], who describe the information loss of non-clustered and isolated ecosystems with increasing level of aggregation. Kandziora et al. [44] compared different land use-land cover (LULC) datasets for ES mapping and found that provisioning services (crop and fodder production) were overestimated when the assessment was based on coarser CORINE LULC data compared to ATKIS and combined ATKIS/InVeKoS/Landsat datasets. Divergent results were largely caused by the inclusion of different land cover classes present in the input datasets.

The effect of changes in spatial resolution is known in geography as the “Modifiable Areal Unit Problem” (MAUP), consisting of a “zoning” effect and an “aggregation” effect, which are caused by changing the extent and grain size, respectively. Changing the resolution and extent but also moving the area under consideration is called the “modifiable” unit and has been shown to affect results [45]. Turner et al. [27] investigated such effects on landscape patterns and demonstrated that with increasing grain size, the landscape becomes more homogeneous, and rare land cover types characterized by a high level of dispersion and small patch size disappear in a non-linear manner. When aggregating spatial data, information loss can thus cause coarse-resolution data to become more homogeneous with reduced variance, thus modifying average values per spatial unit [46]. Qi and Wu [47] also analyzed the effect of changes in scale on landscape patterns but focused on spatial structure, demonstrating their variations related to changes in resolution: autocorrelation describing the spatial structure decreased with increasing level of aggregation until the spatial structure can no longer be detected and the size of the spatial pattern is smaller than the grain size [48]. Typically, autocorrelation decreases with increasing distance because “near things are more related to each other than distant things,” as described in Tobler’s first law of Geography [23].

In this study, we examined the effect of increasing the spatial resolution of model input data on ES estimates across different ES, case studies and models. We focused on mountainous areas because, unlike the lowlands, they feature compressed topography and vertical gradients, creating isolation and leading to sharp social-ecological transitions that are particularly susceptible to global change [49]. We quantified differences in ES estimates when mapping them at comparable coarse and fine resolution at three study sites in Europe (250 m vs. 25 m) and one study site in the U.S. (300 m vs. 30 m). To illustrate the effect of changing spatial resolution on estimates of different ES, we selected agricultural and timber production (provisioning services), flood regulation and carbon sequestration (regulating services), and scenic beauty (cultural service) from the main MA [50] categories. We describe the spatial pattern of ES estimates at different resolutions using Moran’s I correlograms and link differences between fine and coarse resolution ES maps to terrain and model properties. We close with scale-related recommendations for mapping ES to make the resulting maps more comparable, and suggest a four-step approach to address the issue of scale when mapping ES that can deliver information to support ES-based decision making with greater accuracy and reliability.

## Methods

We selected four case studies in mountain regions in Europe (Davos, Trentino and Stubai) and the United States (Puyallup River watershed). Typically for mountain regions, settlements are located at lower elevation and with increasing elevation, agricultural fields and pastures are replaced by forest then alpine tundra and rock, snow, and ice. The comparison of resolution differences in ES mapping at several sites can yield more general and more robust conclusions about scales effects [33].

### Case studies

The **Puyallup River watershed** covers an area of 2,455 km^2^ between longitude 122 28’ to 121 22’ E and latitude 46 47’ to 47 20’ N, near the southern end of the Puget Sound, a glacially carved inlet of the Pacific Ocean in the U.S. state of Washington. The Puyallup River’s headwaters are located on the glaciated western slope of Mt. Rainier (4,392 m a.s.l.), a volcano and the region’s highest mountain. The major port city of Tacoma (population 202,010) lies at the confluence of the Puyallup River and the Puget Sound. The upper watershed remain largely forested, while agricultural land use occurs in valleys and developed land mostly at lower elevations nearer to the Puget Sound.

**Davos** is a mountain town located in the Eastern parts of the Swiss Alps with 11,000 permanent inhabitants and up to 28,000 seasonal tourists; its municipality covers an area of 254.5 km^2^ between longitude 9 43’ to 9 57’ E and latitude 46 40’ to 467 51’ N. It is surrounded by peaks reaching over 3,000 m a.s.l. The main valley is oriented in a NE-SW direction at an elevation between 1,400 and 1,600 m a.s.l. Agricultural fields spread along the valleys. On steeper terrain, land is used for extensive farming and summer pastures. Forests extend up to about 2,100 m a.s.l.

The **Stubai** Valley, including the municipalities of Neustift in Stubai Valley and Fulpmes, is located in the Central Eastern Alps in Austria between longitude 11 6’ to 11 25’ E and latitude 46 55’ to 47 15’ N. It has a permanent population of 8,782 and attracts 1.6 million overnight stays per year. The main valley is oriented to the NE-SW and altitude ranges from 920 to about 3,500 m a.s.l, covering an area of 266 km^2^. The landscape is dominated by forests and grasslands, which differ in management intensity and include meadows of high land-use intensity in the valley bottom, alpine meadows of low land-use intensity and pastures at higher altitudes, along with abandoned pastures and meadows.

**Trentino** is an Alpine region located in Northeast Italy between longitude 10 27’ to 11 57’ E and latitude 45 41’ to 46 28’ N with 530,000 inhabitants. It covers 6,207 km^2^ and most of the area is mountainous, with elevation ranging from about 65 to 3,760 m a.s.l. About 60% of the land is covered by forests, and orchards and vineyards are commonly found on the main valley floors and favorably oriented slopes.

**Figure 1:**
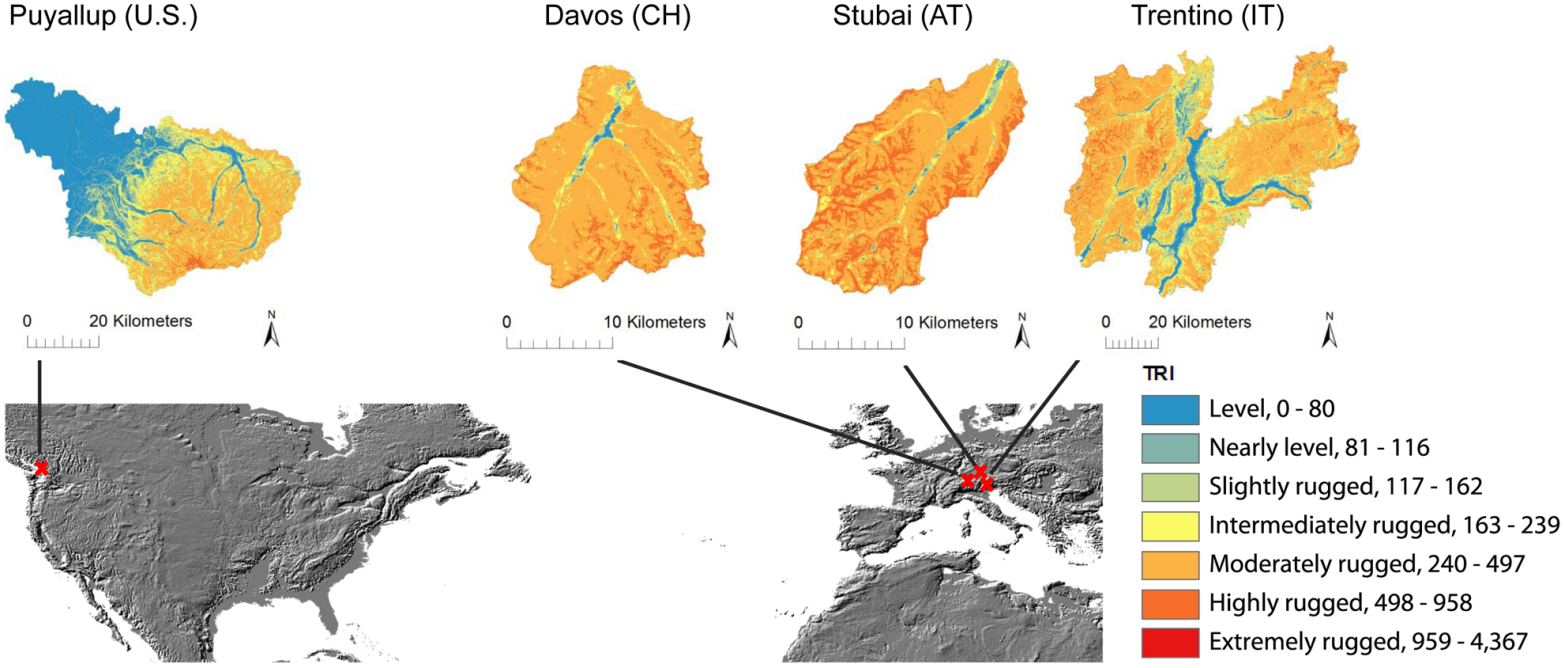
TRI values and location of case study areas (world map: [103]).

To compare the topographical characteristics of the different sites, we calculated the topographic ruggedness index (TRI) [51] using fine-resolution digital elevation model datasets (30 m resolution for the U.S. and 25 m for the European case studies) as a measure of elevation difference between adjacent cells. We categorized TRI into seven classes as suggested by Riley [51] (Fig. 1). Greater TRI indicates a more rugged surface with greater topographic heterogeneity. While Stubai and Davos are mainly covered by mountainous terrain with elevations no lower than 920 and 1,400 m.a.s.l. respectively, Trentino includes larger valleys with gently increasing slopes, and Puyallup covers low-TRI areas near Puget Sound as well as highly rugged terrain around Mt. Rainer.

### Models

A variety of modeling approaches can be used for ES assessment. Martínez-Harms and Balvanera [14] distinguished five categories: (a) assigning constant ES values to each land cover class (lookup tables), (b) expert-based ranking of an environmental variable that influences ES supply, (c) developing “causal relationships” between environmental variables and the supply of ES based on secondary data, (d) extrapolating ES values from primary data such as field measurements to the total study area, (e) regression models, which quantify the relationship between field measurements of ES (response variable) and measurements of environmental variables (explanatory variable).

In this study, we used causal-relationship models to quantify and map the five selected ES. Detailed model properties are described below. In Puyallup, scenic beauty and flood regulation were estimated using the ARIES mapping tool [52]. For the other ES and case studies, LULC and other environmental parameters were linked to secondary data, except the scenic beauty model applied in Davos and Stubai, which is based on a regression model. For each ES and site, we always used the same model with both coarse and fine resolution input data. Models are designed to account for local conditions at different sites, and input data used are of different level of detail depending on data availability as described below. Where possible, models were applied at more than one site (see S3 Table for a list of input data used for each model). Monetary values for ES are expressed in 2012 U.S. dollars. For the U.S. case studies, values were converted to 2012 U.S. dollars using the U.S. Bureau of Labor Statistics CPI inflation calculator. For Davos, Swiss Francs were converted using the historical exchange rate [53]: 1 CHF = 1.1 USD. For Stubai and Trentino the exchange rate was 1 EUR = 1.3 USD [53].

#### Agricultural Production

To estimate agricultural production values, we combined agricultural production statistics with LULC information that identified the spatial extent of agricultural land. The level of detail of production statistics differed between study sites. We did not consider management costs. For the Puyallup case study, agricultural production includes all agricultural products sold – both crops and animals. For Stubai and Davos, only forage production was considered because all parcels are currently managed as intensive, extensive or summer pastures. In Trentino, agricultural products that were valued included tree farming (fruits, olives, nuts), grassland, crop and potato cultivation, and market gardening products.

*Davos, Stubai:* In Davos and Stubai, all parcels are managed as intensive or extensive pastures or, at higher elevation, as summer pastures with cattle grazing during the summer. Only summer pastures were considered in Davos. Forage is either cut as hay or directly grazed by the cattle. We estimated maximum potential forage quantity for each land cover class based on the growing season length, which depends on elevation and productivity of the grassland type. We derived actual forage quantity from potential forage quantity by additionally considering slope, aspect, and summer precipitation. Local topographic parameters (slope, aspect) influence heat balance due to diminished solar radiation. For instance, forage quantity is reduced up to 20% on northern slopes over 10° [54]. The total amount of summer precipitation (April–September) can also be a limiting factor, as increased precipitation yields an increase in forage production. We used fodder price at different elevations and for different production intensities for valuation [55].

*Trentino:* Our analysis included 27 types of agricultural products. Selling prices of agricultural products and their productivity were obtained from existing inventories [56]. We valued productivity using selling prices and mapped the result across agriculture cadastral parcels. Further details are described in Ferrari and Geneletti [57].

*Puyallup:* We summed agricultural land area mapped by LULC datasets by U.S. county. We then divided the total market value of agricultural products sold within each county by actual estimates of the area of farmland in each county from the 2007 U.S. Department of Agriculture – Census of Agriculture [58]. This yielded per-hectare agricultural values for the Puyallup watershed; we applied these values to farmland mapped by fine and coarse resolution LULC datasets.

#### Timber Production

To estimate timber production values per hectare and year, we used timber harvest statistics that were combined with LULC information. We did not consider management costs.

*Stubai*: Based on spatial data for forest and management types, we monetized the average timber harvest for each forest and management type by an average value of $128 per m^3^ of harvested timber [59].

*Davos*: We derived harvestable timber from total forest area at different elevations. Valuation was based on the average annual value of harvested timber [60], which we monetized using an average price of 86 CHF/m^3^ for different timber products. The price considers the amount of timber products sold on the market: 77% construction timber at 100 CHF/m^3^, 8% pulpwood at 55 CHF/m^3^ and 15% firewood at 30 CHF/m^3^ (see [61] for details).

*Trentino:* We derived harvestable timber from forest management plans in the region, with forests divided into parcels of minimum area of 20 hectares. For each parcel, the harvested volume of wood is monitored, as is the volume available for cutting. We multiplied selling prices by the timber harvest and mapped the results. Further details are described in [57].

*Puyallup:* Timber harvest data from the Washington Department of Natural Resources [62] include both the spatial extent of parcels undergoing timber management and timber market data. Timber was valued at $321 per thousand board-feet [63]. At the coarse resolution, we applied this value towards the timber harvest using forest cover change between the 2005–2006 and 2009 GlobCover datasets. At the fine resolution, we applied this value towards the summed timber harvest value in timber harvest polygons for the Puyallup watershed.

#### Carbon Sequestration

We calculated yearly changes in carbon stocks considering above- and belowground biomass, based on available data such as carbon stocks in different vegetation types. All carbon sequestration values were based on Tol’s estimate of $43/ton social cost of carbon as cited in Nelson et al. [7].

*Stubai:* Carbon storage calculations were based on above- and belowground biomass [64]. We derived yearly changes to the carbon stock values from the total biomass of timber stocks [65], [66] and spatial information on forest type, forest structure and management plans for different years.

*Davos*: Quantification of carbon sequestration was based on average yearly growth rate of the forest in Davos [61]. We calculated the corresponding carbon sequestration capacity following Thürig and Schmid [67]. We estimated growth of belowground biomass and its associated carbon stock using appropriate “root to shoot” ratios [68].

*Trentino*: We mapped carbon stock and yearly carbon sequestration based on data for eight forest types [69] and for agriculture (further details in [70]).

*Puyallup:* We paired global and local LULC data with tables that quantify carbon sequestration by landcover type. These include estimates for the U.S. Pacific Northwest for forests [71]–[73], wetland carbon sequestration estimates for mineral soil wetlands in the coterminous United States [71], and cropland and grassland/shrubland estimates for the Marine West Coast Forest region [72]. For forested landcover types, we assumed forests to be of moderate age (65 years). Regionally appropriate carbon estimates were not available for urban land cover, so we underestimate carbon sequestration for this land cover type [74].

#### Scenic Beauty

Ecosystems and their spatial patterns contribute to landscapes of particular beauty, inspiring spiritual, aesthetic values and historic memory. Views of these aesthetically appealing natural landscapes are often valued by residents and tourists [75]. To map the scenic beauty of a landscape, we calculated the visibility of particularly beautiful spots such as mountains, open water, forests, and heterogeneous landscapes. We standardized results between 0 and 100 to yield quantitative, comparable results, which we did not monetize.

*Davos, Stubai:* The model was based on randomly selected viewpoints distributed along roads and hiking paths. We considered fore- and background visibility (i.e., the presence of nearby and distant visual features) by adding mean vegetation and building heights to the ground elevation then calculating the viewsheds. Based on a set of 60 landscape metrics describing area, patch, edge and shape properties, which were summarized using principal component analysis (PCA), we characterized the spatial structure of visible landscapes. Stakeholder preferences were evaluated using a perception study that presented a questionnaire with photographs and asked tourists and residents of the Central Alps to score the photographs according to their scenic beauty. After georeferencing the photographs and calculating the landscape metrics of the related viewpoint, a stepwise linear regression analysis was performed, enabling the quantification of scenic beauty of any viewpoint [76].

*Trentino:* We used a GIS viewshed analysis to calculate the visibility of landscape points of particular beauty for a maximum distance of 10 km, considering the effects of the terrain's surface. Landscape points of particular beauty included natural and cultivated ecosystems, characteristic landscape elements, and archaeological sites; visibility depends on terrain features that may obstruct views. We considered a total of 333 viewpoints, consistent with existing inventories [77].

*Puyallup:* We quantified scenic beauty using Bayesian models that quantified the relative quality of high-quality views (e.g., mountains and open water) and “visual blight“ features that degrade view quality (e.g., developed land), based on a review of the hedonic valuation literature for viewsheds [78]. We mapped beneficiaries of the viewshed analysis as homeowner locations where views enhance property values. We linked beneficiary locations with a viewshed model to map areas that provide scenic views to beneficiaries and how obstructions or visual blight degrade high-quality views [74].

#### Flood Regulation

Methods used to estimate flood regulation capacity differed slightly for each case study, but in all cases we estimated mitigated runoff as the difference between total input precipitation and runoff. Runoff is influenced by factors such as soil properties, vegetation and slope. We did not monetize results.

*Stubai*: We predicted surface runoff using an ordinary least-squares (OLS) regression equation developed based on field experiments at the study site using a rain simulator. All independent variables were tested for multicollinearity. The regression equation used surface runoff as the dependent variable and aboveground phytomass (included as dry weight (gm^−2^) of a 0.3 m x 0.3 m square, harvested at peak biomass), skeleton fraction-soil stone content in 0–0.1 m soil depth (analyzed by sieving disturbed soil samples and weighing the gravel fraction>2 mm), precipitation and elevation as independent variables [79]. We then estimated mitigated runoff of a storm event as the difference between precipitation and runoff.

*Davos*: We used the KINematic Runoff and EROSion (KINEROS) surface runoff model available in the Automated Geospatial Watershed Assessment (AGWA) GIS tool v2.0. KINEROS calculates runoff and peak flow based on terrain, land use and soil data, as well as storm event precipitation [80]. We calibrated peak runoff with rain gauge measurements. We calculated mitigated runoff by subtracting the modeled runoff from total input precipitation.

*Trentino*: We used precipitation data and runoff Curve Number (CN) results to quantify flood regulation. The CN is an empirical parameter developed by the U.S. Soil Conservation Service [81], which is used in hydrology to determine the approximate amount of direct runoff from a rainfall event in a particular area. CN is a function of permeability and land use; its value ranges from 30 to 100. Smaller numbers indicate low runoff potential while larger numbers denote increasing runoff potential.

*Puyallup:* In 45 small sub-watersheds within the Puyallup River watershed, we mapped 1) precipitation, 2) outputs of a Bayesian model of vegetation, topographic, and soil influences on ecosystems’ ability to intercept, absorb, or detain flood water, and 3) developed land within the 100-year floodplain as the beneficiary of flood regulation. We quantified flood regulation in each sub-watershed as the percentage of mitigated flood water (intercepted, absorbed, or detained flood water divided by total precipitation) multiplied by the number of beneficiaries at risk of flooding [74].

### Statistics

Statistical analysis was performed using the statistical software “R”, version 3.0.1 [82]. To compare ES estimates mapped at different spatial resolution and across sites, we calculated the arithmetic mean and standard deviation of the estimates for each ES at each site and resolution. We derived percent changes in ES by calculating the difference between coarse and fine resolution estimates divided by coarse resolution values. We tested for significant differences between high- and low-resolution ES estimates using the Wilcoxon rank sum test [83]. To describe the relationship between land use classes and ES estimates, we calculated box-and-whisker plots [84].

To describe autocorrelation, we calculated Moran’s I correlograms [85] in R with the library sp.correlogram of the spdep package including a test for significance. Correlograms show autocorrelation values for certain distance classes or so-called lags. We chose a lag distance of 500 m for generating distance classes. Weights within classes were equal and row-standardized. Using the same lag distance allows the comparison of Moran’s I values of different sites for a particular distance class. Moran’s I can only be calculated with a minimum number of data points at a specific distance, thus it was not always possible to build distance classes of 500 m. Fine-resolution data were subsampled into random point samples with 1/50 of initial data points to enable the calculation of Moran’s I. For timber production and flood regulation service in Trentino, the size of subsamples was set to 1/150 of initial data points because Trentino covers the largest extent of all study sites, leading to a large initial sample size. Scenic beauty values were only subsampled for Trentino due to already small sample sizes in the other case studies.

## Results

### Effect of Resolution on LULC

Differences between fine- and coarse-resolution LULC classes can in part be related to the TRI values. Averaged over each case study site, TRI was lowest in Puyallup (117), intermediate in Trentino (288), and highest in Davos (364) and Stubai (414). Standard deviation was lowest in Davos (107) and similar in all other case studies (136 in Trentino and Puyallup, 138 in Stubai). The mean TRI values represent the different topographic characteristics of the study sites: while Stubai and Davos are mainly covered by moderately and highly rugged terrain with the highest mean TRI, Trentino includes lower-TRI valley bottoms with intermediate TRI values, and Puyallup includes substantial low-TRI areas.

**Figure 2:**
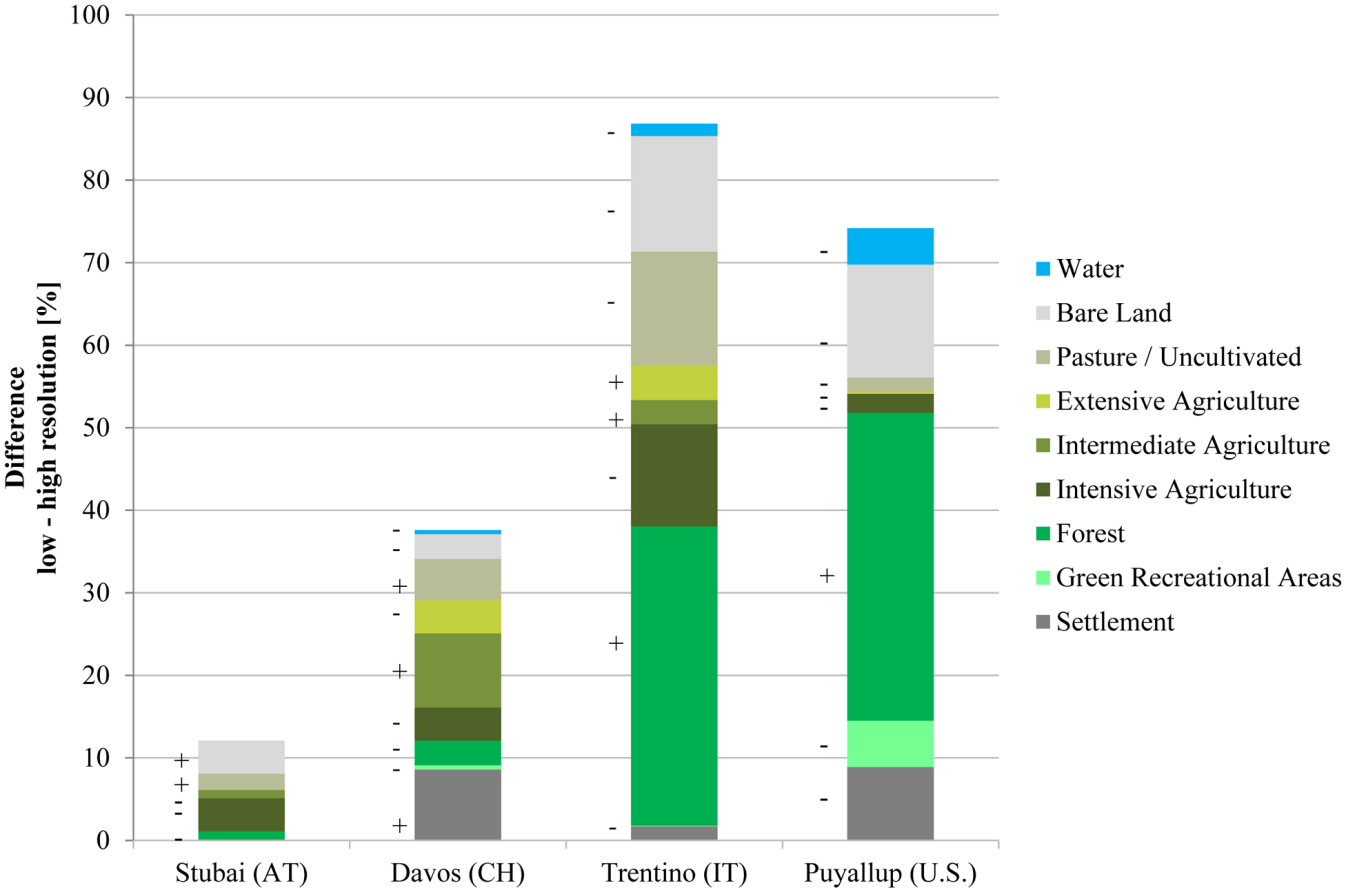
Absolute difference in coverage (%) between fine and coarse resolution LULC classification. + and – indicate over- and underestimation at the coarse resolution.

Differences between fine- and coarse-resolution LULC classes were greatest in the Trentino and Puyallup case studies and were greatest for forests, whose extent was largely overestimated by the coarse-resolution datasets, followed by bare land, which was typically underestimated at the coarse resolution (Fig. 2, S1 Table). Other LULC classes with substantial differences between fine- and coarse-resolution datasets were intensive agricultural and pastures/uncultivated in the Trentino case site and settlement in Puyallup and Davos. LULC classification data in the greater-TRI Davos and Stubai case studies differed less between resolutions. In Puyallup, NLCD LULC datasets were used for the fine resolution analysis and GlobCover for the coarse resolution, whereas in Davos, Stubai and Trentino, CORINE data were used at the coarse resolution and local datasets at the fine resolution. LULC maps derived from satellite images (CORINE, NLCD and GlobCover) contain classification errors which likely differ for classes and regions [21]. In the aggregation process for coarse-resolution datasets, information about dispersed and isolated structures is easily lost while clustered structures are more likely to persist [26].

### Ecosystem Services Maps

**Figure 3:**
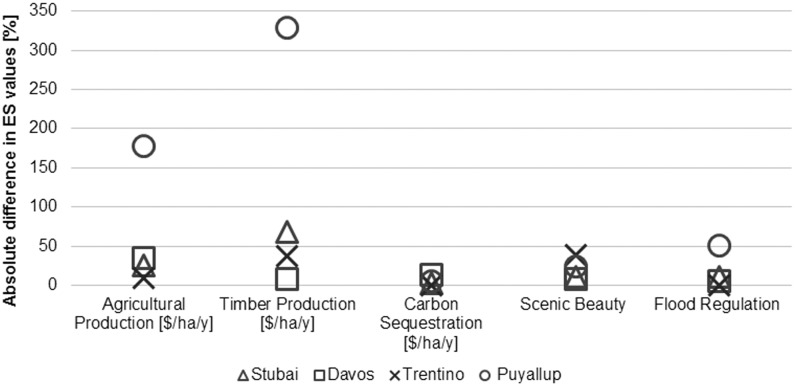
Absolute difference between coarse and fine resolution ES values (%) for all ES and case study areas.

ES estimates vary considerably at different resolutions (Fig. 3); mean values and percentage changes between resolutions are provided in the S2 Table. Maps of all ES as well as LULC maps are provided in S1 File; raster data are provided in S1 Data. Changes in ES estimates at different resolution for agricultural production, timber production, carbon sequestration and flood regulation were greatest in Puyallup and for scenic beauty differences were greatest in Trentino; both areas are characterized by lower TRI than the Stubai or Davos case study sites. Despite this tendency of lower-TRI case study sites to show greater variations in ES estimates at different resolutions, the relationship between terrain ruggedness and changes in ES estimates at different resolution was not linear, i.e., the differences in ES estimates did not constantly decrease with increasing TRI. Notably, agricultural production and flood regulation in Trentino showed little variation between resolutions whereas in Stubai, agricultural and timber production values change considerably at different resolutions.

The highest difference - 329% - between timber production values calculated at the different resolutions in Puyallup is an artifact of a coarse-resolution LULC dataset (GlobCover), which dramatically overestimated the extent of forest when compared to high-resolution NLCD data. Similarly, the CORINE LULC classification did not distinguish between intensive and extensive pastures, which led to greater agricultural production estimates at the coarse resolution in Stubai. Because agricultural and timber production services were both estimated with causal-relationship models, the changes in ES values at different resolutions were mainly related to differences in the LULC datasets and their classifications. Differences between fine- and coarse-resolution carbon sequestration estimates were smallest for all sites and models compared to the other ES types, except in Davos where differences in timber production, flood regulation and scenic beauty estimates were lower. Carbon sequestration in Davos was estimated using data for forest growth rates, another measure that is sensitive to differences in forest land cover classification.

Changes in scenic beauty estimates between resolutions were greatest in Trentino, followed by Puyallup and were lowest in Davos and Stubai. In Trentino and Puyallup, differences between fine and coarse resolution ES estimates were strongly related to how the viewpoints were selected. The number of use points representing the demand was substantially lower in the coarse-resolution dataset in Puyallup, which led to large differences in ES estimates at the different resolutions. For mapping flood regulation, demand for the service was also quantified in Puyallup, resulting in an overestimate of developed land located in floodplains when using coarse-resolution data, leading to a 51% overestimate of this service. In this case, the differences in ES estimates between resolutions can be related to the ARIES mapping approach (which considers spatially separated supply of and demand for ES) as well as the LULC classifications that over- or underestimated the number of use points for scenic beauty and flood regulation. In contrast, while different flood regulation mapping approaches were applied in the European case studies, the differences between coarse and fine resolution flood regulation ES estimates were minor as these approaches did not account for demand for ES.

### Effect of Resolution on ES Spatial Patterns

**Figure 4:**
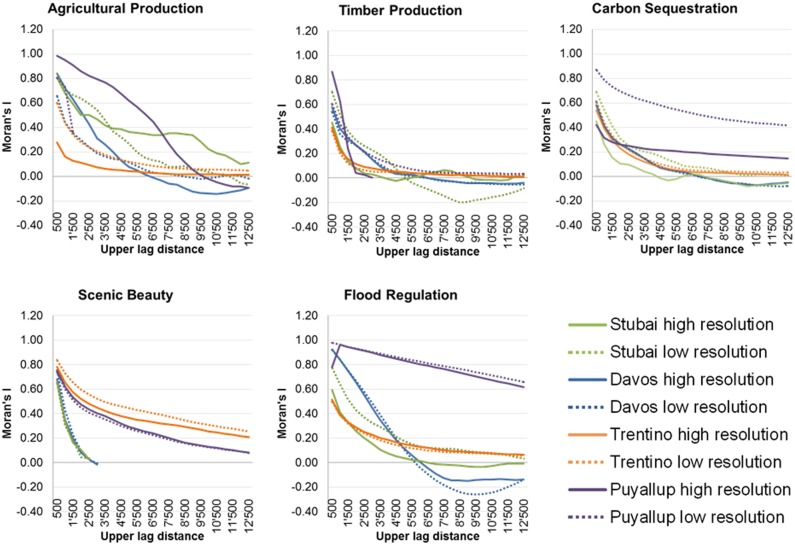
Moran’s I for all case study areas and ES at fine and coarse resolution plotted against lag distance classes of 500 m. Indicated on the x-Axis is the upper distance of each lag.

Fig. 4 shows Moran’s I correlograms for fine- and coarse-resolution ES estimates for the different case studies. Moran’s I values range from −1 to 1, where 1 describes a highly clustered (correlated) pattern. Autocorrelation quantifies the lack of independence (e.g., correlation) between features located near to each other. With increasing distance, this relationship weakens and autocorrelation becomes insignificant as Moran’s I approaches zero. Increasingly negative values of I indicate greater dispersion, with a value of −1 indicating perfect, regular dispersion. Lacking an adequate number of data points for scenic beauty in Davos and Stubai and agricultural production in Puyallup, Moran’s I was only calculated for some lags for these case studies and ES. All ES in all study sites showed positive spatial autocorrelation, which decreased nonlinearly with distance. Spatial patterns of all ES values changed at different resolutions: as aggregation increases, fine-resolution variance was lost and patterns became more homogeneous, with more abrupt transitions occurring between patches (see S1 File). In highly heterogeneous mountain environments, abrupt changes in spatial pattern such as valleys can lower autocorrelation values because spatial patterns persist over shorter distances than in more homogeneous landscapes. These mountain-region gradients persist through the aggregation process, leading to similar spatial patterns at fine and coarse resolution as opposed to more homogeneous terrain, where aggregation produces greater changes in the spatial patterns. In fact, autocorrelation tended to be greatest across all distance classes in Puyallup (the site with the lowest mean TRI) for agricultural production, timber production, carbon sequestration and flood regulation. For scenic beauty, the greatest autocorrelation was found in Trentino followed by Puyallup.

Lag distances generated by Moran’s I correlograms in Fig. 4 give more information about changes in spatial patterns between fine- and coarse–resolution estimates. Timber production, for example, is highly dependent on LULC, but the highly clustered structure at low distances decreased rapidly and leveled off at a lag distance of 4,500 m at all sites (even if the areas of forests differed greatly between fine and coarse resolution), indicating that the spatial pattern is not highly related to the total forested areas in the case study sites.

For carbon sequestration at the fine resolution in Stubai, different forest and management types were considered in the analysis, leading to a more dispersed pattern when compared to the low resolution analysis. Similarly, clustering of carbon sequestration values is greater in Puyallup at the high resolution whereas in Davos and Trentino, the spatial structure varied little between scales.

For agricultural production, autocorrelation decreased gradually with increasing lag distance and the spatial pattern between fine- and coarse-resolution analyses varied across all sites, indicating different patterns of agricultural production values. Agriculture in all mountain-region case studies is generally located in the lowlands and valley bottoms. In comparison to the European sites, where farmland is divided into small parcels, agricultural fields in the Puyallup watershed are located in broader, flatter valley bottoms; parcels there are aggregated to larger patches with greater autocorrelation values.

Scenic beauty and flood regulation showed similar spatial structures between fine- and coarse-resolution assessments across all sites, with greatest scale differences in Stubai for flood regulation. Flood regulation was quantified by sub-watershed in all studies except Trentino, resulting in coarse structures that were similar for both scales but generating different ES estimates. In Trentino, the structure was very dispersed at both resolutions; it was highly clustered with a linear decrease in Puyallup. For scenic beauty in Davos and Stubai, autocorrelation rapidly decreased until a lag distance of about 2,500 m. Scenic beauty values at these sites were derived based on viewpoints along roads. The Moran’s I correlograms thus reflect the linear, clustered structure of the roads. The estimates derived by viewshed analysis in Puyallup and Trentino showed greater clustering but very similar patterns across resolutions as presented in S1 File.

### Model Properties and Influence of LULC Classification

Due to different levels of data availability and the need to account for site-specific conditions, selected mapping approaches included different parameters (e.g., elevation, LULC) at different levels of detail (e.g., number of LULC classes). To isolate the effects of scale from those caused by differing level of detail of the models, we compared agricultural production, timber production and carbon sequestration estimates derived from simple relationships between LULC and secondary data, when using the same LULC classes across all case study sites. Flood regulation and scenic beauty could not reliably be determined by such a simple relationship because they are more complex processes (i.e., flood regulation) or because they reflect unique regional preferences (i.e., scenic beauty). We selected LULC classes based on the least common denominator, which reduced the number of LULC classes to seven: forest, grassland, agriculture, bare land, water, wetlands and settlement. Comparing these estimates to those generated using the site-specific approaches described above, scale differences in carbon sequestration were 34% and 58% greater in Trentino and Puyallup, respectively, but were similar in Davos and Stubai (1% reduced in Stubai). Differences between high and low resolution timber production were lower (Stubai −63%, Puyallup −95%) or similar (Davos +2%, Trentino +6%) when using lookup tables and common LULC classes. Agricultural production showed greater scale differences when using the simpler approach in Stubai (+11%) and Trentino (+34%) and lesser scale differences in Davos (-30%) and Puyallup (-642%). Because of the few LULC classes used in this simpler approach, the difference between fine- and coarse-resolution mapping was directly related to the difference in the LULC classification. The more LULC classes were considered in the site-specific approach, the greater the difference found when using the simple approach. Lesser differences indicated that the more complex site-specific approach relied on LULC with a similar classification.

**Figure 5:**
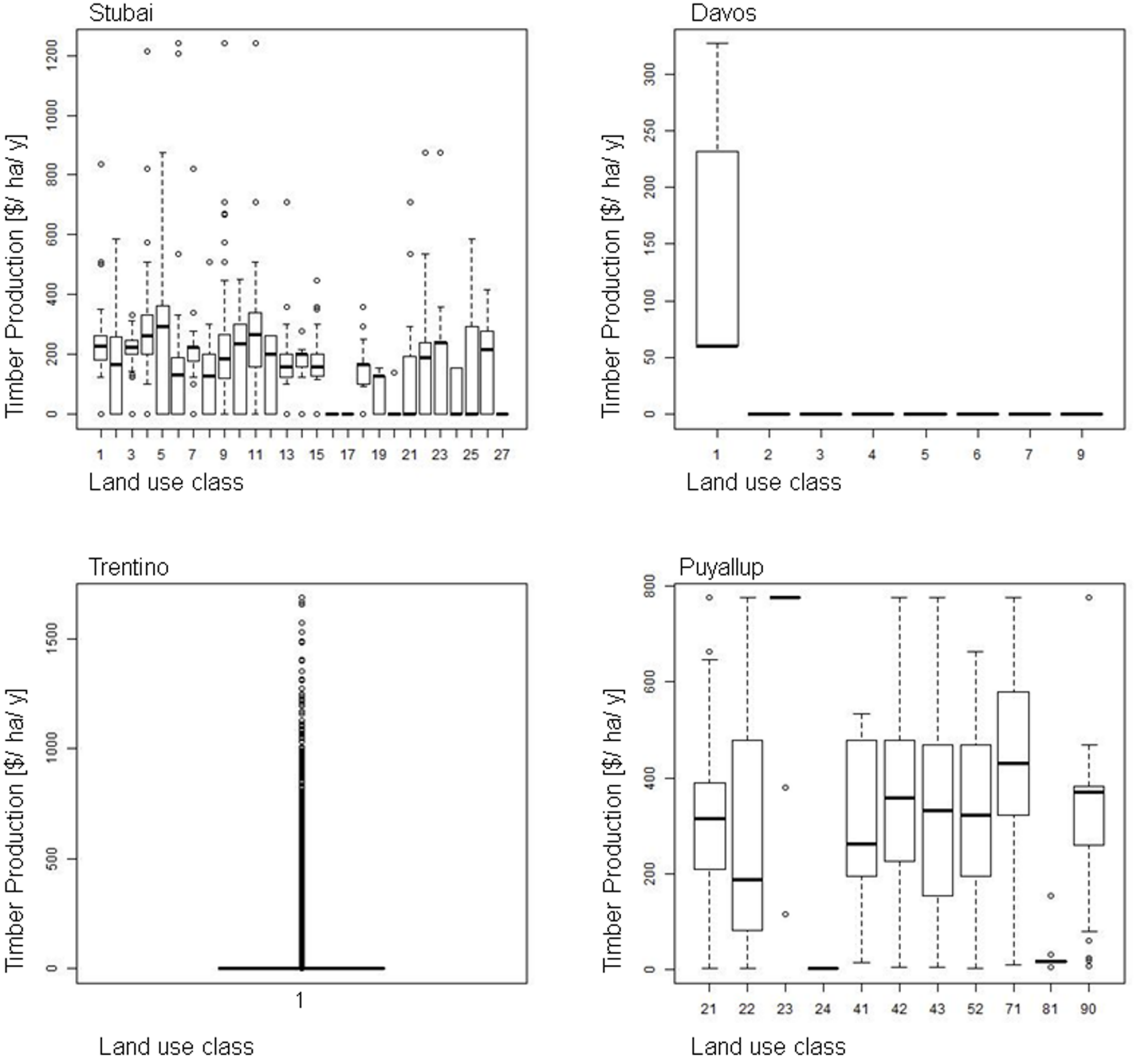
Box- and Whisker plots of land use classes (x-Axis) and ES values (y-Axis) for fine resolution timber production.

**Figure 6:**
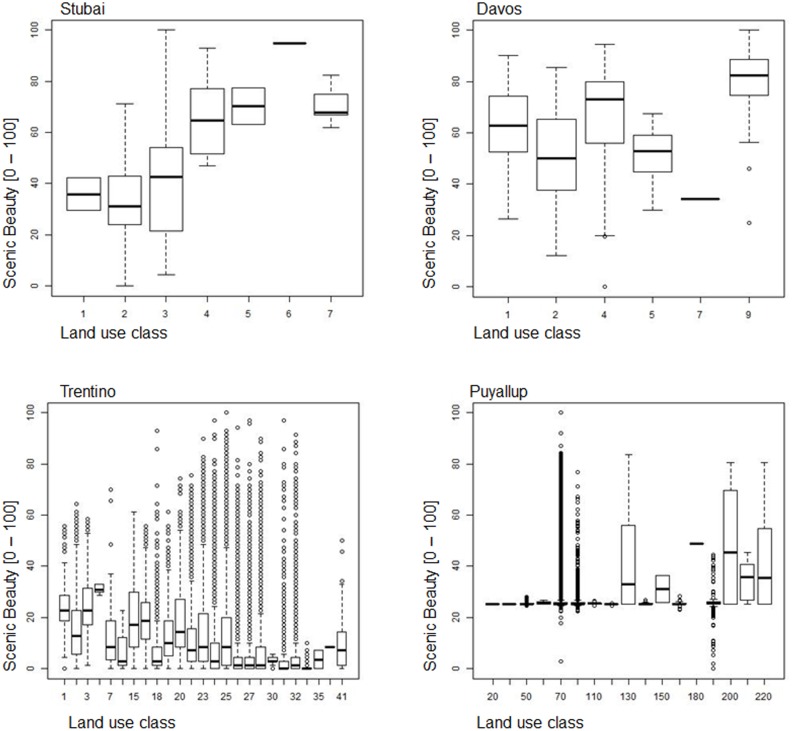
Box- and Whisker plots of land use classes (x-Axis) and ES values (y-Axis) for coarse resolution scenic beauty across all sites.

All models use LULC data as input data, but they differ in the level of detail (e.g., different forest or pasture types); some models use additional biophysical and/or socio-economic data to quantify and map ES (see S3 Table). The specificity or generality in the various LULC classifications shown in Fig. 2 influenced ES estimates, particularly when the model strongly depended on a particular level of specificity in its LULC classification. Some services were modeled using several different classes, while others mainly relied on one or a few land use classes. Figs. 5 and 6 show the relationship between LULC classes and ES values where different LULC classes were plotted on the x-axis and ES estimates on the y-axis. The ES models rely on different numbers of LULC classes to different extents. A smaller spread of the boxplot for a certain land use class indicates a direct link between that LULC class and the service, e.g., a production value is assigned to a certain crop. A greater spread of the boxplot within an individual land use class indicates that other parameters may be influential (e.g., elevation) causing a wider spread of ES estimates. Timber production in Davos and Trentino, for example, was modeled as fully depending on the “forest” class at the fine resolution. Yet ES values differed because productivity was estimated at different elevation levels (i.e., other input data helped determine final ES values aside from LULC). Differences in input values of these additional parameters between fine and coarse resolution add to the LULC differences in the final ES estimate. In addition to the spread of the boxplot of an individual LULC class, the dependency of ES estimates on LULC is related to the number of classes considered. Where many classes were present, the effect of a single class was reduced and the connection between land use and ES was less pronounced, as seen for example in fine resolution timber production and coarse resolution scenic beauty in Trentino.

## Discussion

EU Member States are required to map ES by 2014 as stated under Target 2, Action 5 of the Biodiversity Strategy [86]. Similarly, various U.S. government agencies are beginning to work on quantifying, valuing, and mapping ES [87] resulting for example in the recent release of the first “EnviroAtlas,” which delivers 250 ES-related data layers to users ([88]) (and in a proliferation of modeling and mapping approaches [89]. Due to the different approaches (including different choice of scales, model complexity, data inputs, and output metrics) used in the different countries, the comparability of results across administrative boundaries will be difficult [17]. If better knowledge about the effects of up- and downscaling of mapped ES estimates were available, decision makers could better identify priority areas and policy measures at aggregated levels, such as the European level [18].

Mapped ES for four different mountainous case studies at two different resolutions using similar mapping approaches showed significant differences in ES estimates across scales. For some services such as carbon sequestration, scale differences were minor across all case study sites. In contrast, the other services showed differences between fine and coarse resolution estimates of up to 329%. Differences in ES estimates at different resolutions can be related to terrain properties. The spatial pattern analysis showed that the sharper the gradients, the more consistent spatial pattern is observed between resolutions: in lower-TRI regions with smoother biophysical gradients, more information on the spatial pattern of ES estimates was lost with changes in resolution. Our results showed that changes in ES values were greatest in Puyallup for all services except scenic beauty, where differences were greatest in Trentino (both case study sites characterized by a lower TRI values). Furthermore, changes in the spatial pattern between resolutions tended to be greatest for the lower-TRI case study sites - Puyallup and Trentino - except for flood regulation, where changes were greatest in Stubai. This seems to confirm that compressed topography and vertical gradients lead to more isolated social-ecological systems, where mountain biophysical systems share a long-term co-evolution with humans ­ an interdependency that results in tightly coupled but highly diverse mountain social-ecological systems [90].

However, the effect of increasing differences in ES estimates in lower-TRI areas was not consistent across the four case studies, although there was a tendency for resolution differences to be greater in lower TRI areas. This inconsistency was caused by the differences in the models applied; in all cases the selected models were adapted to local data and site-specific conditions. The models generally used similar approaches to generate estimates with analogous metrics, but in a multi-continent comparative study, it is likely unrealistic to expect model structure and data inputs to be identical. If a model strongly depended on LULC data, land use classification error, e.g., caused by MAUP, was directly incorporated into the ES estimates. This calls for a “science of scale” in mapping ES, in which scale is included as an explicitly stated variable in the analysis (similar to the call by Meentemeyer and Box [91] for the development of a “science of scale” in ecology), particularly when developing policy strategies in data poor environments or comparing values across sites or countries.

Our analysis used simple statistical measures and spatial autocorrelation metrics to investigate the effect of scales on ES mapping estimates. Yet, there have been a wide range of other landscape metrics used to study scale effects in ecology, remote sensing, and geography in the past three decades, seeking to better understand those landscape features that can be extrapolated or interpolated across spatial scales [91]–[95], some of them reflecting shifts in the average landscape properties about patch size and shape. Both the effects of the shape of the mapping unit and the size of the study area are known to have a strong effect on the outcomes of the assessments [96]. The shape is known to change within-sample unit variance, changing the effects of environmental gradients. The size of the study area may incorporate multiple subregions, each having different underlying social-ecological processes and environmental conditions, which can make values non-stationary over the entire area. But other studies have demonstrated that simple variance and correlation measures, such as the analyses conducted in this paper, were able to detect scale breaks in real and artificial landscapes when calculated at different grain sizes [97], [98]. It is more important, however, to describe the determinants of patterns, thus the mechanisms that generate and maintain them. Since in most cases it is not feasible to run all ES assessments at a fine resolution, ES mapping must thus begin with (1) a thorough understanding of the spatial and temporal relationships between ES patterns and social-ecological processes. Ostrom [99] recommended that the entry point of the analysis of each socio-ecological system be based on the question of major interest to the researcher, user, or policy maker. The MA also highlighted the importance of spending initial time and resources to investigate the mechanisms and processes of the socio-ecological system [42]. Only after knowing that maps and analyses can provide adequate information to answer the original question should the user begin applying spatial analyses and landscape metrics to the input data to better understand up- and downscaling and aggregation of ES maps. The spatial correlation analysis conducted in this study is just a first step in this direction. Getis [100] lists analyses that can assess the spatial nature of the data including e.g., tests on assumptions of spatial stationarity and spatial heterogeneity and measures to understand temporal effects. Similarly Wu [101] lists relevant landscape metrics, which could be tested both on the input data of differing resolution and on ES mapping estimates. Based on such analyses, one can then (2) choose the modeling approach that best fits the question and spatial characteristics of the system property of interest, (3) choose appropriate metrics by considering the heterogeneity that is relevant to the ecological process of interest, and (4) formulate a theoretical relationship between a mapping approach and the socio-ecological processes, improving the likelihood that empirical evidence can be related to the results of the analysis. Multi-scale and cross-scale approaches have been promoted in recent years and are appropriate when the problem or objectives intrinsically require a multi-scale approach, the responses require syntheses of data across scales, analysis of causality and trade-offs are important to users, or a sense of ownership of the assessment is required from stakeholders at various scales [22], [102]. The emerging concept of macrosystem ecology has gone one step further in emphasizing the investigation of the interactions between different temporal and geographic scales (telecoupling) integrating knowledge of different disciplines [33]. Particularly important in all these approaches is their application to multiple landscapes for establishing reliable relationships between landscape-scale pattern and process [22], [101] But for all these approaches, a key is to understand the effect of scales on the unpacking or aggregation of variables up and down a conceptual hierarchy. Generating the “right scale” map requires us thus to first understand how the variables under consideration affect ES interactions, supply, and demand related to the specific scientific or policy question, and to adjust the scale of the study to be as close as possible to that relevant to decision makers.

## Conclusions

By mapping ES for four mountain region case studies in Europe and the U.S., we demonstrate the importance of addressing scale issues when comparing ES maps conducted at different resolutions. The amount of change in the ES estimates with increasing level of aggregation is different for each site, ES, dataset used and model applied. However, the resolution effects are not equivalent for all ES. Differences in ES estimates between resolutions were more pronounced in lower-TRI areas due to the persistence of sharp gradients in greater TRI areas even when using coarse-resolution data. In less rugged terrain or other environments where information about non-clustered, isolated ES is more likely to be lost at coarse resolution, we recommend finer resolution analysis for monitoring of isolated, non-clustered patterns of ES supply and demand. Furthermore, if ES value maps are compared, it is important that they be grounded in a thorough understanding of the relationships between ES patterns and socio-ecological processes. Further research in this area should seek to better understand theoretical relationships between mapping approaches and underlying socio-ecological processes so that the ES-based information will be accurate and reliable enough to support improved decision making.

## Supporting Information

S1 Table LULC by case study area (%) for each scale. S1 (Legend): (x  =  not existing).
(PDF)

S2 Table Mean ES values and change (%) by case study area. S2 (Legend): Not significant differences (α = 0.05) are marked bold.
(PDF)

S3 Table List of input data used for each model.
(PDF)

S1 Data File geodatabase of raster data of ES and TIR.
(RAR)

S1 File Maps of ecosystem services.
(RAR)
