# Supplementary material for: On the Effects of Scale for Ecosystem Services Mapping
Source: PLoS One. 2014 Dec 30;9(12):e112601. doi: 10.1371/journal.pone.0112601 (PMC4280228; doi:10.1371/journal.pone.0112601)
Supplement: S1 Table — LULC by case study area (%) for each scale. S1 (Legend): (x = not existing). (PDF) [file pone.0112601.s001.pdf]

Land use	Stubai (AT) 25m	Stubai (AT) 250m	Davos (CH) 25m	Davos (CH) 250m	Trentino (IT) 25m	Trentino (IT) 250m	Puyallup (U.S.) 30m	Puyallup (U.S.) 300m
Water	x	x	0.7	0.2	2.0	0.5	6.2	1.8
Bare Land	47.0	51.0	39.0	36.0	14.0	x	15.5	1.8
Pasture / Uncultivated	15.0	17.0	29.0	34.0	16.7	2.9	3.8	2.1
Extensive Agriculture	4.0	4.0	4.0	x	0.5	4.7	0.3	x
Intermediate Agriculture	1.0	x	x	9.0	x	2.9	x	x
Intensive Agriculture	4.0	x	4.0	x	14.9	2.5	2.3	0.0
Forest	28.0	27.0	22.0	19.0	46.9	83.1	55.0	92.3
Green Recreational Areas	x	x	0.7	0.2	x	0.1	5.6	x
Settlement	0.9	1.0	0.4	9.0	5.0	3.3	11.0	2.1
