# Supplementary material for: On the Effects of Scale for Ecosystem Services Mapping
Source: PLoS One. 2014 Dec 30;9(12):e112601. doi: 10.1371/journal.pone.0112601 (PMC4280228; doi:10.1371/journal.pone.0112601)
Supplement: S2 Table — Mean ES values and change (%) by case study area. S2 (Legend): Not significant differences (α = 0.05) are marked bold. (PDF) [file pone.0112601.s002.pdf]

Ecosystem Service	Case Study	Mean Value<br>Fine resolution	 Coarse resolution	 Change [%]
Agricultural Production [$/ha/y]	Stubai	1,434	6,363	77
Agricultural Production [$/ha/y]	Davos	2,719	4,157	35
Agricultural Production [$/ha/y]	Trentino	2,698	2,457	-10
Agricultural Production [$/ha/y]	Puyallup	5,643	2,033	-178
Timber Production [$/ha/y]	Stubai	75	229	67
Timber Production [$/ha/y]	Davos	153	157	2
Timber Production [$/ha/y]	Trentino	36	57	37
Timber Production [$/ha/y]	Puyallup	733	180	-329
Carbon Sequestration [$/ha/y]	Stubai	62	64	3
Carbon Sequestration [$/ha/y]	Davos	18	16	-14
Carbon Sequestration [$/ha/y]	Trentino	123	123	0
Carbon Sequestration [$/ha/y]	Puyallup	206	218	5
Scenic Beauty [relative values, 0-100]	Stubai	41	45	**10**
Scenic Beauty [relative values, 0-100]	Davos	73	68	-7
Scenic Beauty [relative values, 0-100]	Trentino	6	9	39
Scenic Beauty [relative values, 0-100]	Puyallup	21	28	24
Flood Regulation [%]	Stubai	51	46	-10
Flood Regulation [%]	Davos	91	87	-5
Flood Regulation [dimensionless scale]	Trentino	53	53	0*
Flood Regulation [relative values]	Puyallup	1,939	3,965	51

* The slight difference between high and low resolution flood regulation and carbon sequestration in Trentino is not represented in the mean, please refer to the maps in Appendix S4
