# Supplementary material for: On the Effects of Scale for Ecosystem Services Mapping
Source: PLoS One. 2014 Dec 30;9(12):e112601. doi: 10.1371/journal.pone.0112601 (PMC4280228; doi:10.1371/journal.pone.0112601)
Supplement: S3 Table — List of input data used for each model. (PDF) [file pone.0112601.s003.pdf]

Ecosystem service	Case study	Input layer	Coarse-resolution data source	Fine-resolution data source
Carbon sequestration	Puyallup	Forest successional stage	No data (Bayesian priors)	Interagency Vegetation Mapping Program (30 m)
		Tree canopy cover	UMD-GLCF (1 km^2)	National Land Cover Dataset (30 m)
		Hardwood: softwood ratio	GlobCover 2005-06 (300 m)	Interagency Vegetation Mapping Program (30 m)
		Summer high: winter low temperature differential	WorldClim (1 km^2)	PRISM (800 m)
	Stubai	Forest parcels	CORINE Land Cover (250 m)	Local datasets Tyrolean Information System (tiris, Land Tirol©) (25 m)
		Yearly forest growth rate	Changes of the timber stock [65,66]	Changes of the timber stock [65,66]
	Davos	Land use type	CORINE Land Cover (250 m)	Local land use-land cover dataset (25 m)
		Growth of belowground biomass	"root to shoot" ratio of belowground to aboveground biomass [68]	"root to shoot" ratio of belowground to aboveground biomass [68]
		Yearly forest growth rate	4.87 m^3/ha/y [60]	4.87 m^3/ha/y [60]
		Carbon mitigation capacity	Following [67]: 5.52 t CO_2/ha/y in Davos	Following [67]: 5.52 t CO_2/ha/y in Davos
	Trentino	Forest parcels	CORINE Land Cover (250 m)	Local forest inventory (25 m)
		Land use type	CORINE Land Cover (250 m)	Local land use-land cover dataset (25 m)

		Value of carbon sequestration	Following a local inventory [69]	Following a local inventory [69]
Flood regulation	Puyallup	Precipitation	WorldClim (1 km^2)	PRISM (800 m)
		Slope	SRTM (90)	National Elevation Dataset (30 m)
		Hydrologic Soils Group	University of Vermont (1 km^2)	SSURGO (30 m)
		Impervious surface cover	NASA (1 km^2)	National Land Cover Dataset (30 m)
		Tree canopy cover	UMD-GLCF (1 km^2)	National Land Cover Dataset (30 m)
		Vegetation type	GlobCover 2005-06 (300 m)	National Land Cover Dataset (30 m)
		Vegetation height	No data (Bayesian priors)	Landfire (30 m)
		Mean days of precipitation/year	No data (Bayesian priors)	PRISM (vector data)
		Forest successional stage	No data (Bayesian priors)	Interagency Vegetation Mapping Program (30 x 30 m)
		Developed land	GlobCover 2005-06 (300 m)	National Land Cover Dataset (30 m)
		Floodplain extents	Used local data for calculating flood extents	FEMA (vector data)
		DEM	SRTM (90 m)	National Elevation Dataset (30 m)
		Hydrography	Used local data for calculating water routing	Washington DNR (vector data)
	Davos	Land use type	CORINE Land Cover (250 m)	Local land use-land cover dataset (25 m)
		Soil data	National soil dataset	Local soil dataset MAB (25 m) for main valleys, joined with national soil dataset for remaining area
		Precipitation	Interpolation map of	Interpolation map of

		(map)	rain gauge measurements (250 m)	rain gauge measurements (25 m)
		Precipitation (observation record)	Precipitation measurement at rain gauge from national meteorological service	Precipitation measurement at rain gauge from national meteorological service
		DEM	SRTM (250 m)	Swisstopo [87] (25 m)
	Stubai	DEM	SRTM (250 m)	Local datasets Tyrolean Information System (tiris, Land Tirol©) (25 m)
		Aboveground phytomass [57] related to land cover	CORINE Land Cover (250 m)	Local LULC dataset (25 m)
		Soil	Skeleton fraction-soil stone content in 0–0.1 m soil depth [79].	Skeleton fraction-soil stone content in 0–0.1 m soil depth [79].
		Precipitation	Average value from national meteorological service; own measurements	Average value from national meteorological service; own measurements
	Trentino	Curve Number	Hydrological parameter assessing the capacity of the land (as a function of the cover types and of the permeability of the subsoil) to retain rain-off [81]	Hydrological parameter assessing the capacity of the land (as a function of the cover types and of the permeability of the subsoil) to retain rain-off [81]
			Vector map; 1:10000	Vector map; 1:10000
Scenic beauty	Puyallup	Elevation	SRTM (90 m)	National Elevation Dataset (30 m)
		Lakes and oceans	GlobCover 2005-06 (300 m)	National Land Cover Dataset (30 m)

		Developed land	GlobCover 2005-06 (300 m)	National Land Cover Dataset (30 m)
		Clearcuts	No data (Bayesian priors)	Washington DNR (vector data)
		Highways	Digital Chart of the World (vector data)	TIGER (vector data)
		Residential land use	GlobCover 2005-06 (300 m)	National Land Cover Dataset (30 m)
	Davos<br>Stubai	Viewpoints	120 points randomly distributed along roads and hiking paths outside of forest and settlements	120 points randomly distributed along roads and hiking paths outside of forest and settlements
		Land use	CORINE Land Cover (250 m)	Local LULC dataset (25 m)
		Terrain	SRTM (250 m)	Swisstopo / Tyrolean Information System (tiris, Land Tirol©) (25 m)
		Background	SRTM (1 km)	SRTM (1 km)
		Questionnaire	Details in [76]	Details in [76]
	Trentino	Points of landscape beauty	333 points surveyed by [77]	333 points surveyed by [69]
		Terrain	SRTM (250 m)	Local DTM (25 m)
Timber production	Puyallup	Timber parcels	GlobCover 2005-06 (300 m)	Washington DNR
	Davos	Forest parcels	CORINE Land Cover (250 m)	Local LULC dataset (25 m)
		Elevation	SRTM (250 m)	Swisstopo (25 m)
		Harvestable amount of timber	Observed yearly amount between 1995 and 2005 [60]	Observed yearly amount between 1995 and 2005 [60]
	Stubai	Forest parcels	CORINE Land Cover	Local datasets Tyrolean Information

			(250 m)	System (tiris, Land Tirol©) (25 m)
		Harvesting method	CORINE Land Cover (250 m)	Local dataset including harvesting methods (25 m)
	Trentino	Forest parcels	CORINE Land Cover (250 m)	Local forest inventory (25 m)
Agriculture production	Puyallup	Agricultural land use	GlobCover 2005-06 (300 m)	National Land Cover Dataset (30 m)
		Market value of agricultural products	2007 USDA Census of Agriculture [58]	2007 USDA Census of Agriculture [58]
	Davos Stubai	DEM	SRTM (250 m)	Swisstopo / Tyrolean Information System (tiris, Land Tirol©) (25 m)
		Agricultural land use	CORINE Land Cover (250 m)	Local LULC dataset (25 m)
		Summer precipitation	Average value from national meteorological service	Average value from national meteorological service
		Fodder price	Market prices reported in yearly surveys	Market prices reported in yearly surveys
		Agricultural land use	CORINE Land Cover (250 m)	Local LULC dataset (25 m)
	Trentino	Agricultural products and fodder price	Market prices reported in yearly surveys [56]	Market prices reported in yearly surveys [56]
